# Burnout among pharmacy professionals in Qatar: A cross-sectional study

**DOI:** 10.1371/journal.pone.0267438

**Published:** 2022-05-05

**Authors:** Yassin Eltorki, Oraib Abdallah, Sadaf Riaz, Sara Mahmoud, Mohamed Saad, Nosyba Ez-Eldeen, AbdulAhad Ashraf, Eman Al-Hamoud, Noriya Al-Khuzaei, Suhaila Ghuloum

**Affiliations:** 1 Pharmacy Department, Mental Health Services, Hamad Medical Corporation, Doha, Qatar; 2 Pharmacy Department, Al-Wakra Hospital, Hamad Medical Corporation, Doha, Qatar; 3 Pharmacy Department, Hamad bin Khalifa Medical City, Hamad Medical Corporation, Doha, Qatar; 4 Psychiatry Department, Mental Health Services, Hamad Medical Corporation, Doha, Qatar; 5 Psychiatry Department, Weill Cornell Medicine—Qatar, Doha, Qatar; Imam Abdulrahman Bin Faisal University, SAUDI ARABIA

## Abstract

**Background:**

Pharmacists’ roles and responsibilities have expanded in the modern pharmacy profession, and the expectations from pharmacists have increased. This has been associated with new psychological challenges and emotional stress that can induce burnout.

**Objective:**

To determine the prevalence of burnout syndrome and factors associated with burnout among pharmacy professionals in the healthcare system in Qatar.

**Methods:**

This institutional-based cross-sectional study was conducted on 850 pharmacy professionals within Hamad Medical Corporation (HMC) in Qatar. Convenience sampling was followed. The survey utilized the Maslach Burnout Inventory (MBI) Toolkit™ for Medical Personnel and a modified version of the Astudillo and Mendinueta questionnaire. Statistical analyses were performed using Stata version 16 for Windows and SAS Studio 3.8 (Enterprise Edition). P-value of less than 0.05 was considered significant.

**Results:**

One hundred ninety-four pharmacy professionals (23%) responded to the survey. The prevalence of burnout was 19.7% [95% Confidence interval (CI); 13.8% - 26.8%] among 142 respondents who completed MBI questionnaire and 17.3% [95% CI; 11.7%-24.2%] among 139 respondents who completed Astudillo Mendinueta questionnaire. The most commonly reported factors that may lead to burnout were: tension and lack of organization in teamwork (59.6%), lack of recognition of or indifference to effort from patients, superiors, and colleagues (58.2%), and demanding and challenging patients and family members (56.7%). Multiple regression analysis showed that overtime working hours per month is independently associated with a higher risk of burnout [odds ratio (OR), 1.57; 95% CI, 1.15–2.14 for each 10-hours increase in monthly overtime, P = 0.005], while non-Arab ethnicity is associated with lower risk of burnout [OR, 0.27; 95% CI, 0.1–0.75; P = 0.012].

**Conclusions:**

There is a relatively low prevalence of burnout syndrome among health-system pharmacy professionals in Qatar. Overtime working hours and Arab ethnicity are independently associated with burnout.

## Introduction

Pharmacists play a vital role in today’s hospital environment, being part of a multidisciplinary team for patient care [1]. Their role in evaluating prescriptions has led to an increased identification and resolution of medication errors and increased medication therapy optimization [2–4]. Hospital pharmacists embrace pharmaceutical care by combining specialized therapeutic knowledge, experience, and judgment with traditional drug-oriented services, aiming for optimizing patient outcomes. The expansion of pharmacists’ role and increased day-to-day responsibilities and expectations of them have been associated with new psychological challenges and emotional stress that can induce burnout [5, 6].

Burnout is defined as "a psychological syndrome of emotional exhaustion (EE), depersonalization (DP), and reduced personal accomplishment (PA) that can occur among individuals who work with other people in some capacity" [7]. According to the International Classification of Disease-11 (ICD-11), burnout syndrome is conceptualized as an occupational phenomenon, not a mental health illness. However, reports show that burnout may often be mistaken for depression, with which it shares common features [8]. It starts with emotional exhaustion, physical depletion, and emotional strain, then morphs into reduced personal accomplishment and feelings of incompetence. Left unmanaged, it progresses into depersonalization; an attempt to put distance between oneself and service recipients by actively ignoring the qualities that make them unique and engaging to people [9, 10].

There is clear evidence of burnout among pharmacists worldwide. In the United States, a study conducted in 2016 among hospital clinical pharmacists revealed high levels of burnout (61.2%) [1]. In France, a survey of community pharmacists detected burnout syndrome in 56.2% of respondents; 10.5% of whom presented with a severe syndrome. Severe burnout syndrome was considered if the participants had high EE, high DP, and low PA [11]. Similar high prevalence of burnout was reported among hospital pharmacists in Australia and Japan [6, 12]. Regionally, the studies are scarce and limited to community pharmacists only. A 2007 survey of Turkish community pharmacists revealed that 27.1% had moderate levels of emotional exhaustion [13]. Several studies on burnout among physicians, nurses, medical students, and combined populations of healthcare workers have been undertaken in the Middle East [14]. However, we found little to no papers that study burnout among pharmacy professionals in particular [15].

Qatar National Health Strategy (2018–2022) identifies mental health and well-being as one of seven priority areas. The strategy states that "Mental health and well-being enables people to be more actively engaged in society, work productively and contribute to their communities" [16]. Hamad Medical Corporation (HMC) is the principal public healthcare provider in Qatar which manages several hospitals that are accredited by The Joint Commission International (JCI). Pharmacy professionals in HMC collaborate with physicians and other health care providers to ensure safe and effective use of medications, enabling patients to achieve positive therapy outcomes and minimize medication-related problems. The compliance with the JCI accreditation has put extra pressure on pharmacists and other health care providers to ensure the highest standards of patient care. HMC has a policy that recommends carrying out stress risk assessment and mitigating workplace stress whenever possible. This study focuses on the occupational health of pharmacy professionals.

We aimed to identify the prevalence and symptoms of burnout syndrome and to explore factors independently associated with burnout syndrome among pharmacy professionals in HMC, Qatar.

## Materials and methods

### Study design and setting

This was a quantitative cross-sectional questionnaire-based study; conducted within HMC, the primary provider of secondary and tertiary healthcare in Qatar. HMC was formed in 1979 and manages 13 health facilities as well as the National Ambulance Services and Home Health Care Services. HMC is considered Qatar’s leading healthcare provider for more than two million people living and working in the country. At the time of the study, there were around 850 pharmacy professionals employed in HMC. The survey was circulated to HMC pharmacy professionals from June to August 2020.

### Study sample and sampling technique

Convenience sampling was followed. All pharmacy designations, including pharmacy technicians and their seniors, pharmacists (including junior pharmacists, staff pharmacists, and senior pharmacists), pharmacy supervisors, clinical pharmacists, clinical pharmacy specialists, pharmacy assistant directors, and pharmacy directors within HMC were eligible to participate. Community pharmacists or others working in governmental health care centers were excluded. The study survey was distributed via a web-based link to 850 pharmacy professionals. Single population proportion formula with correction for finite population was used to calculate sample size. Assuming 50% prevalence of burnout among pharmacy professionals, as per previous studies [1, 6, 12], a minimum of 143 responses was required to give a margin of error of ± 7.5% with a 95% confidence interval for the prevalence of burnout. Based on previous surveys conducted in HMC pharmacy, we anticipated a response rate of around 25%, yielding an expected 210 responses which is above the minimum required sample size (143 responses).

### Survey instrument

We developed an anonymous, self-administered questionnaire using Survey Monkey, and circulated the link from the corporate pharmacy office to eligible participants via email. The questionnaire was divided into four sections: (1) Socio-demographic data (2) Maslach Burnout Toolkit™ for Medical Personnel (MBI-MP) [17] (3) Causes of burnout (4) Symptoms of burnout syndrome using Astudillo and Mendinueta questionnaire [18].

The MBI survey is a twenty-two self-assessment questionnaire utilized to detect the presence of burnout syndrome. The MBI-MP is the standardized tool used in research to identify and determine the magnitude of burnout syndrome in medical personnel and is applicable to healthcare professionals broadly. The questionnaire is divided into nine questions assessing EE, five on DP, and eight on reduced PA. Participants were asked to rank each response from 0–6 according to how often they feel them. Responses were categorized into three tiers (low, moderate, or high) as follows: EE, low (0–16), moderate (17–26), and high (≥27); DP, low (0–6), moderate (7–12) and high (≥13), and PA, low (≤31), moderate (32–38) and high (≥39). Burnout was defined by the updated Maslach-recommended criteria of "high EE and high DP" or "high EE and low PA" [19].

Astudillo and Mendinueta is a 19-items questionnaire that describes the manifestations of burnout. It was first described by Wilson Astudillo and Carmen Mendinueta in their article about exhaustion syndrome in palliative care [18]. It has been used in studies about burnout, including in Qatar [20, 21].

Participants were asked to choose how often they have been exposed to each item (never, sometimes, often, always). The sum of the scores was calculated with a total minimum score of 0 to a maximum score of 57. Participants who scored more than 23 were considered to have burn out [20].

Research team members validated the draft questionnaire for appropriateness, language, clarity and flow of questions and some changes were made. The questionnaire was then piloted in a convenience sample of 10–15 pharmacy professionals who were excluded from the final respondents. This was to check for clarity, readability, and comprehensiveness of all items. Minor modifications were subsequently made in the sociodemographic section only.

### Data analysis

Descriptive statistics were used to describe respondents’ demographics and professional characteristics. Categorical variables were expressed as frequencies with percentages and compared using Chi square test (or Fisher’s exact test when more than 20% of cross-tabulation cells had expected frequencies < 5 or minimum expected frequency was < 1), ordinal variables as medians with interquartile ranges and compared using Wilcoxon rank sum test, and continuous variables as means with standard deviations and compared using t-test. Prevalence was computed with the 95% confidence interval. Multiple logistic regression model was used to examine the effect of demographic and professional characteristics on the incidence of burnout among respondents. Backward elimination of variables with p-value > 0.1 for the likelihood ratio test was used to select the final model. Firth’s penalized likelihood estimation was used due to quasi-complete separation. Lack of fit was assessed using Hosmer-Lemeshow test. Effects of different variables were presented as odds ratios with the corresponding 95% confidence intervals. P-values of <0.05 were considered statistically significant. Statistical analyses were performed using Stata version 16 for Windows and SAS Studio 3.8 (Enterprise Edition).

### Ethics approval

The study received ethical approval from HMC Institutional Review Board (IRB) (MRC-01-19-520). An invitation was sent to all pharmacy professionals to participate in the study along with an information sheet. Proceeding to complete the survey after reading the online information was indicative of informed consent. Participation was voluntary, participants could withdraw at any time, and data was reported and analyzed anonymously. The study was conducted in accordance with the Helsinki Declaration.

## Results

### Characteristics of respondents

The survey was sent by email to 850 pharmacy practitioners, with four subsequent reminders (S1 Fig). Over twelve weeks, 194 pharmacy practitioners responded to the survey, giving a response rate of 23%. Of the respondents, 102 (52.6%) were female, the mean age was 35.8±7.2 years, and 115 (59.3%) were of Arabic ethnicity. Half of the respondents held a post-graduate degree (Master of Science, Doctor of Philosophy, Doctor of Pharmacy or Board of Pharmacy Specialties) or joined a pharmacy residency program, and the majority were in the intermediate job grade group. Half of the participants worked shift duties, and more than one-third worked as on-call pharmacists. Other characteristics of respondents are shown in Tables 1 and 2.

**Table 1:** Sociodemographic characteristics of all respondents (n = 194).

Characteristics	Responses, n (%)
Age in years, mean [SD]	35.8 (7.2)
Ethnicity	
Non-Arab	79 (40.7)
Arab	115 (59.3)
Female	102 (52.6)
Currently pregnant	3 (1.5)
Marital Status	
Single	45 (23.2)
Married	145 (74.7)
Divorced	4 (2.1)
Children, median (IQR)	2 (0–3)
Sole breadwinner	110 (56.7)
Living with family	166 (85.6)
Smoking	
Never	168 (86.6)
Former	14 (7.2)
Current	12 (6.2)
Alcohol use	3 (1.5)

**Table 2:** Educational and occupational characteristics of all respondents (n = 194).

Characteristics	Responses, n (%)
Acquiring additional degrees	
No	110 (56.7)
Master of Science	40 (20.6)
Doctor of Pharmacy	16 (8.2)
Doctor of Philosophy	4 (2.1)
Residency program	7 (3.6)
Other	17 (8.8)
Years of experience since graduation	
Less than 2	19 (9.8)
2–5	26 (13.4)
6–10	41 (21.1)
11–15	47 (24.2)
16–20	41 (21.1)
More than 20	20 (10.3)
Years of experience in HMC	
Less than 2	48 (24.7)
2–5	42 (21.6)
6–10	62 (32)
11–15	28 (14.4)
16–20	8 (4.1)
More than 20	6 (3.1)
Job grade group	
Low	28 (14.4)
Intermediate	160 (82.5)
High	6 (3.1)
Place of work	
General	113 (58.2)
Supply chain	8 (4.1)
Clinical	65 (33.5)
Administration	8 (4.1)
Area of practice	
General	150 (77.3)
Oncology	13 (6.7)
Mental health	10 (5.2)
Critical Care/ED	21 (10.8)
Working shift hours	97 (50)
Working on-call	75 (38.7)
Working hours per week	
40	108 (55.7)
41–50	53 (27.3)
51–60	20 (10.3)
61–70	5 (2.6)
More than 70	8 (4.1)
Overtime hours per month	
Less than 10	81 (41.8)
10–20	46 (23.7)
21–30	25 (12.9)
31–40	19 (9.8)
More than 40	23 (11.9)
Number of patients per day	
0–5	12 (6.2)
6–10	11 (5.7)
11–15	11 (5.7)
16–20	23 (11.9)
More than 20	137 (70.6)

ED: Emergency Department; HMC: Hamad Medical Corporation.

### Prevalence of burnout among respondents

**Fig 1:**
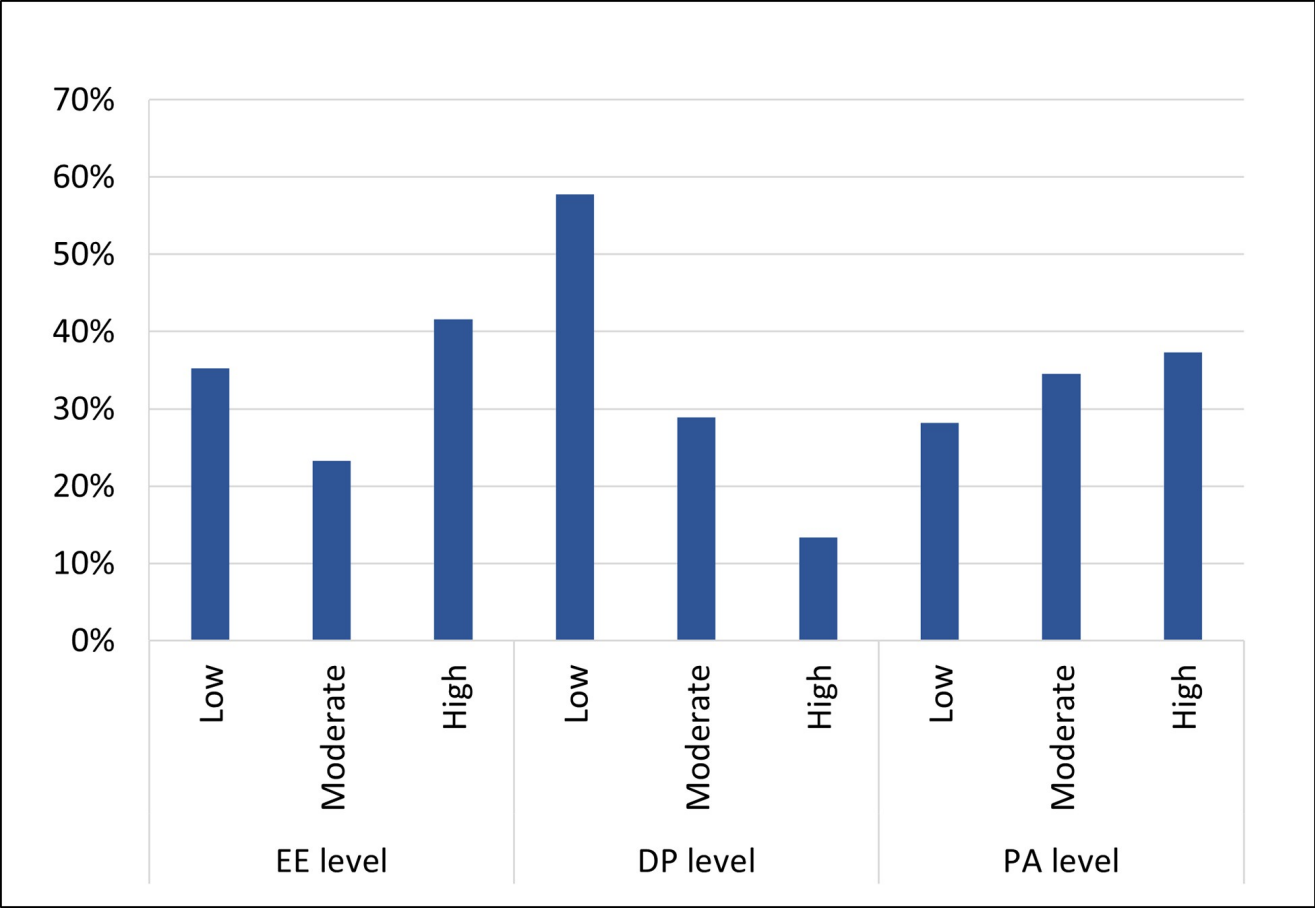
Levels of the components of the MBI inventory among respondents (n = 142). DP: depersonalization, EE: emotional exhaustion, PA: personal accomplishment.

One hundred and forty-two respondents (73%) completed the MBI score. The total scores of the MBI inventory components: emotional exhaustion, depersonalization, and personal accomplishment, had median (interquartile range) values of 22 (11–35), 6 (2–11), and 36 (30–42) respectively. Fig 1 displays the levels of the MBI inventory components categorized as low, intermediate, and high. Based on the criteria previously defined in the methods section, scores of 28 respondents (19.7%) were consistent with burnout. Tables 3 and 4 compare the characteristics of respondents with MBI results consistent with burnout to the remaining respondents. The two groups were similar except for ethnicity and overtime hours per month. One hundred and thirty-nine completed the Astudillo Mendinueta score, of whom 24 (17.3%) had scores consistent with burnout as defined previously in the methods. The median number of burnout symptoms reported was 15 (interquartile range; 9–21 symptoms).

**Table 3:** Comparison of sociodemographic characteristics of respondents with and without burnout according to MBI score (n = 142).

Characteristic	MBI score consistent with burnout, n (%)		P-value
	No	Yes	
	n = 114	n = 28	
Age in years, mean (SD)	35.8 (7.9)	34.4 (5.9)	0.366
Ethnicity			0.007
Arab	62 (54.4)	23 (82.1)	
Non-Arab	52 (45.6)	5 (17.9)	
Female	63 (55.3)	18 (64.3)	0.387
Currently pregnant	3 (2.6)	0 (0)	1*
Marital Status			0.902*
Single	28 (24.6)	8 (28.6)	
Married	83 (72.8)	20 (71.4)	
Divorced	3 (2.6)	0 (0)	
Children, median (IQR)	2 (0–3)	1 (0–3)	0.587
Sole breadwinner	63 (55.3)	14 (50)	0.616
Living with family	97 (85.1)	25 (89.3)	0.765*
Smoking			0.551*
Never	101 (88.6)	23 (82.1)	
Former	7 (6.1)	3 (10.7)	
Current	6 (5.3)	2 (7.1)	
Alcohol use	2 (1.8)	0 (0)	1*

* Fisher’s exact test was used.

**Table 4:** Comparison of educational and occupational characteristics of respondents with and without burnout according to MBI score (n = 142).

Characteristic	MBI score consistent with burnout, n (%)		P-value
	No	Yes	
	n = 114	n = 28	
Acquiring additional degrees			0.4*
No	69 (60.5)	15 (53.6)	
Master of Science	22 (19.3)	4 (14.3)	
Doctor of Pharmacy	6 (5.3)	4 (14.3)	
Doctor of Philosophy	3 (2.6)	0 (0)	
Residency program	6 (5.3)	1 (3.6)	
Other	8 (7)	4 (14.3)	
Years of experience since graduation			0.123*
Less than 2	14 (12.3)	2 (7.1)	
2–5	16 (14)	5 (17.9)	
6–10	29 (25.4)	5 (17.9)	
11–15	19 (16.7)	11 (39.3)	
16–20	24 (21.1)	2 (7.1)	
More than 20	12 (10.5)	3 (10.7)	
Years of experience in HMC			0.952*
Less than 2	27 (23.7)	7 (25)	
2–5	27 (23.7)	6 (21.4)	
6–10	34 (29.8)	10 (35.7)	
11–15	18 (15.8)	5 (17.9)	
16–20	5 (4.4)	0 (0)	
More than 20	3 (2.6)	0 (0)	
Grade group			0.693*
Low	12 (10.5)	4 (14.3)	
Intermediate	98 (86)	24 (85.7)	
High	4 (3.5)	0 (0)	
Place of work			0.588*
General	68 (59.6)	16 (57.1)	
Supply chain	4 (3.5)	1 (3.6)	
Clinical	35 (30.7)	11 (39.3)	
Administration	7 (6.1)	0 (0)	
Area of practice			0.268*
General	85 (74.6)	24 (85.7)	
Oncology	9 (7.9)	2 (7.1)	
Mental health	7 (6.1)	2 (7.1)	
Critical care/ED	13 (11.4)	0 (0)	
Working shift hours	54 (47.4)	14 (50)	0.802
Working on-call	43 (37.7)	8 (28.6)	0.366
Working hours per week			0.633*
40	63 (55.3)	15 (53.6)	
41–50	31 (27.2)	7 (25)	
51–60	13 (11.4)	5 (17.9)	
61–70	2 (1.8)	1 (3.6)	
More than 70	5 (4.4)	0 (0)	
Overtime hours per month			0.017*
Less than 10	56 (49.1)	7 (25)	
10–20	26 (22.8)	7 (25)	
21–30	17 (14.9)	3 (10.7)	
31–40	8 (7)	7 (25)	
More than 40	7 (6.1)	4 (14.3)	
Number of patients per day			0.55*
0–5	10 (8.8)	1 (3.6)	
6–10	6 (5.3)	2 (7.1)	
11–15	9 (7.9)	1 (3.6)	
16–20	10 (8.8)	5 (17.9)	
More than 20	79 (69.3)	19 (67.9)	

ED: Emergency Department; HMC: Hamad Medical Corporation.

* Fisher’s exact test was used.

The prevalence of burnout was 19.7% (95% Confidence interval [CI]; 13.8% - 26.8%) among 142 respondents who completed MBI questionnaire and 17.3% (95% CI; 11.7%-24.2%) among 139 respondents who completed Astudillo Mendinueta questionnaire. Among 139 respondents who completed both questionnaires, the presence of burnout was indicated by both in 113 (81.3%) responses, while the remaining 26 (18.7%) responses had discordant results (Table 5). Cohen’s Kappa statistic of agreement between the criteria of burnout based on the two scores was 0.386 which indicates week agreement.

**Table 5:** Agreement between MBI and Astudillo Mendinueta criteria (n = 139).

Burnout score		Burnout present based on Astudillo Mendinueta criteria		Total
		No	Yes	
Burnout present based on MBI criteria	No	100 (71.9%)	11 (7.9%)	111 (79.9%)
	Yes	15 (10.8%)	13 (9.4%)	28 (20.1%)
Total		115 (82.7%)	24 (17.3%)	139

### Causes and predictors of burnout among respondents

One hundred forty-one respondents reported their perceived causes of burnout. The most commonly reported causes were tension and lack of organization in teamwork (59.6%), followed by lack of recognition of or indifference to effort from patients, superiors, and colleagues (58.2%) then demanding and challenging patients and family members (56.7%) (Table 6).

**Table 6:** Factors that respondents believe may contribute to burnout (n = 141).

Factor	n (%)
Tension and lack of organization in teamwork	84 (59.6)
Lack of recognition of or indifference to effort from patients, superiors, and colleagues	82 (58.2)
Demanding and challenging patients and family members	80 (56.7)
Inadequate working conditions—medical or personal or insufficient time	68 (48.2)
Facing emergency situations and work overload in the daily routine	64 (45.4)
Continuous contact with human suffering	52 (36.9)
Giving care to terminal patients	49 (34.8)
Lack of preparation in dealing with medical and spiritual problems	42 (29.8)
Need to develop services to complement medical care	42 (29.8)
Working in new or unknown services	41 (29.1)
Other	15 (10.6)

Multiple regression analysis showed that overtime hours per month is independently associated with a higher risk of burnout [odds ratio (OR), 1.57; 95% CI, 1.15–2.14 for each 10-hours increase in monthly overtime, P = 0.005] while a non-Arab ethnicity is associated with lower risk of burnout [OR, 0.27; 95% CI, 0.1–0.75; P = 0.012] (Table 7).

**Table 7:** Predictors of MBI-defined burnout among pharmacy practitioners.

Characteristic	Initial model		Final model*	
	aOR (95% CI)	p value	aOR (95% CI)	p value
Age (years)	0.94 (0.8–1.11)	0.485		
Female	2.84 (0.77–10.56)	0.118		
Ethnicity				
Arab (reference group)	1.00	-	1.00	-
Non-Arab	0.27 (0.08–0.93)	**0.038**	0.27 (0.10–0.75)	0.012
Current pregnancy	0.19 (0.004–9.16)	0.403		
Marital status				
Single (reference group)	1.00	-		
Married	1.25 (0.39–4.03)	0.703		
Divorced	0.74 (0.02–26)	0.868		
Smoking status				
Non-smoker (reference group)	1.00	-		
Current smoker	4.04 (0.28–57.36)	0.303		
Former smoker	6.01 (0.82–44.31)	0.078		
Alcohol use	0.14 (0.001–23.45)	0.453		
Living with Family	0.95 (0.20–4.50)	0.944		
Sole bread winner	0.55 (0.18–1.68)	0.297		
Number of patients per day	0.98 (0.58–1.65)	0.927		
Place of work				
General (reference group)	1.00	-		
Administration office	0.28 (0.005–16.71)	0.540		
Clinical pharmacy service	3.18 (0.74–13.64)	0.119		
Supply chain	9.27(0.48–179.46)	0.141		
Area of practice				
General (reference group)	1.00	-		
Critical care/ED	0.07 (0.002–1.78)	0.107		
Oncology	0.54 (0.09–3.23)	0.497		
Mental health	2.61 (0.37–18.54)	0.339		
Working as shifts	1.89 (0.53–6.70)	0.325		
Working as on-call	0.63 (0.19–2.02)	0.433		
Working hours per week	1.04 (0.62–1.74)	0.885		
Acquiring an additional degree	1.26 (0.44–3.63)	0.663		
Overtime hours per month	1.73 (1.15–2.60)	**0.008**	1.57 (1.15–2.14)	0.005
Experience in HMC	0.55 (0.26–1.17)	0.119		
Experience since graduation	2.22 (0.80–6.20)	0.126		
Designation by grade	0.69 (0.12–4.06)	0.682		

ED: Emergency department; CI: Confidence interval; HMC: Hamad Medical Corporation; aOR: adjusted odds ratio.

* Hosmer-Lemeshow test indicated adequate model fit (p value = 0.429).

## Discussion

In the current study, we found that about 20% of pharmacy professionals in Hamad Medical Corporation demonstrated consistent signs of burnout syndrome. Additionally, we found that Arab descent and increased overtime hours per month are independently associated with burnout syndrome among pharmacy professionals. To our knowledge, this is the first burnout assessment study directed towards health-system pharmacy professionals practicing in the state of Qatar.

The prevalence of burnout syndrome among health-system pharmacy professionals in Qatar contrasts the reported higher prevalence of burnout among pharmacists regionally and worldwide. A survey-based study done in 2016 in The United States among hospital clinical pharmacists, with 974 responses and 11.4% response rate, revealed high prevalence of burnout (61.2%). It was most common among females who have been in the pharmacy profession for almost eight years, ranking them as the highest among any medical specialty. Emotional Exhaustion (EE) was found to be the primary driver of burnout [1]. Similarly, in France, a nationwide cross-sectional survey study done among the French community pharmacies in 2015, which received 1,339 responses, revealed that burnout syndrome was detected in 56.2% of respondents; 10.5% of them presented with severe burnout. Severe burnout syndrome was considered if the participants had high EE, high DP, and low PA. Factors identified to be associated with severe burnout syndrome were male gender, large urban areas, and working time. Depression and anxiety were found in 15.7% and 42.4% of respondents, respectively [11]. Another study aimed to investigate psychological distress, burnout, and the associated risk factors in hospital pharmacists in Japan reported a high prevalence of psychological distress and burnout (49.2%) [6].

In the Middle East context, there are several studies on burnout among physicians, nurses, medical students, and combined populations of healthcare workers which reveal similar global trends of high burnout among healthcare professionals [14]. In the current study, almost 41% of the respondents experienced high emotional exhaustion and 13% experienced high depersonalization. Unlike in Qatar, pharmacists in Saudi Arabia showed 25% emotional exhaustion and 55.9% depersonalization [22]. The lower prevalence in depersonalization in Qatar may be explained by the difference in prevalence of personal achievement which is 37% in Qatar versus 15% in Saudi Arabia. The inverse correlation between personal achievement and depersonalization is consistent with previous research by Maslach et al. [23].

Multiple risk factors are known to be associated with burnout. In our study, survey results show that the top factors associated with increased risk of burnout were lack of organization, insufficient recognition, and lack of personal time or work-life balance. A study by Jones et al. comprehensively described the risk factors for developing burnout syndrome. These factors include: younger age, financial constraints, having children, spending more than 50 hours per week working, having too many students or residents, being less satisfied with career growth, having too many non-clinical duties, being involved in a longitudinal training program, having difficult coworkers, being intellectually challenged or spending less time in professional development [1].

Gender may also have an effect on the prevalence of burnout. In our analysis, females had a slightly higher risk of burnout but this was not statistically significant. A study by El-Ibiary et al. suggested that female gender is associated with a forty percent increased risk of emotional exhaustion among faculty members [24].

Our analysis shows no difference in the prevalence of burnout between pharmacy professionals with regards to marital status, living conditions, or having children. Similar results are seen in a study by Alharbi et al. conducted in Saudi Arabia [22]. Previous data suggest that having children younger than twelve years of age is associated with a high risk of depersonalization [24]. A systematic review reports that having young children or no children is associated with a higher rate of burnout and emotional exhaustion, respectively [25].

The impact of post-graduate degree or training on burnout syndrome is debatable. Our study showed that acquiring an additional degree was not associated with a higher risk of burnout. In other studies, pharmacists who hold postgraduate degrees with scholarly and teaching responsibilities are more inclined to develop burnout, especially if they have an official academic posting [24, 26]. Being a pharmacy resident, especially PGY2 specialty residency, is associated with high risk of burnout [26]. In contrast, Durham et al. showed no difference in the incidence of burnout among pharmacists who completed residency or fellowship programs [27]. In our study, it was not possible to evaluate the impact of completing the residency program since the number of pharmacy residents participating in the study was too small (n = 7).

Literature review suggests that work-related factors are far more influential than completing post-graduate training on burnout. These factors include overtime hours, number of patients, role clarity, and leadership robustness [10]. Our data show that the total number of overtime hours is positively correlated with an increased risk of burnout syndrome, particularly when overtime hours exceeds 30 hours per month. This is consistent with other studies among pharmacists and other healthcare professionals where 50 working hours or more was most associated with a higher risk of burnout syndrome [26].

Published studies indicate that pharmacists in an early stage of their career are more subject to burnout compared to those with at least fifteen years of experience [25, 27]. In our study, the highest risk of burnout was seen among pharmacy professionals with less than five years of experience and those with eleven to fifteen years of experience. However, the sample size in these subgroups was too small to demonstrate a statistical significance. Nonetheless, it was obvious that all pharmacists with fifteen years of experience or more had significantly less risk of burnout which is consistent with the literature evidence.

The area of specialty also seems to have an impact on the incidence of burnout. In our study, pharmacy professionals practicing in the General Medicine department demonstrated a slightly higher risk of burnout (74.6% vs. 85.7%) compared to other specialties such as oncology and mental health. This could be due to a higher patient-to-pharmacist ratio in General Medicine. A study by Smith et al. shows that a patient-to-pharmacist ratio greater than 25:1 was significantly correlated with a higher risk of burnout [26]. Pharmacists practicing in critical care or emergency departments demonstrated resilience where none of them reported burnout syndrome, despite working in a highly dynamic and demanding environment. This contrasts with published research which shows at least 50% burnout among critical care pharmacists, especially those working in highly specialized centers [26]. Our finding could be explained by having reasonable pharmacist-patient ratio, and working less overtime hours per month due to the absence of on-call system in these services.

Finally, the country of origin is an important factor that affects burnout in the state of Qatar. This finding is unique to this study. It is evident that burnout syndrome is less prevalent among non-Arab pharmacists versus Arabs practicing at Hamad Medical Corporation. The exact reason for the difference is not well known. It could mean that some communities form stronger support groups compared to others. In all other reviewed studies, burnout did not differ according to the country of origin [27]. However, most of those studies were conducted on Caucasian respondents and mostly in North America [28].

To avoid burnout, it is recommended to promote self-care and personal well-being by maintaining a work-life balance. Shanafelt et al. recommended the following leadership strategies to prevent burnout [29]: periodical assessment of burnout, selection of influential leaders, development of anti-burnout interventions, cultivation of a healthy community at work, utilizing rewards, aligning values and strengthening culture, promotion of work-life integration, provision of resilience resources, and funding research dedicated to burnout [30].

To our knowledge, this is the first study to address burnout among health-system pharmacy professionals in Qatar. Measuring burnout using two separate scoring systems for the prevalence and symptoms of burnout is an important strength in our research. The major limitation of the study is the response rate where about 23% only participated (194 out of 850); this affects the representativeness of the sample and the generalizability of the results. Another limitation is that we were unable to study the effect of social factors on stress such as exercise, leadership, and social interactions. We also did not inquire about any previous diagnosis of depression which has overlapping symptoms with burnout syndrome. However, inquiry about depression might not yield accurate responses due to the high level of mental health stigma in The Middle East.

We suggest that more studies should be conducted in the Middle East with a special focus on the cultural and social factors that affect burnout. Future studies should evaluate the association of burnout with the incidence of pharmacy-related medication errors. More studies are needed to examine these finding.

## Conclusion

The prevalence of burnout syndrome among health-system pharmacy professionals in Qatar is about 20%, which is lower than its prevalence among pharmacists regionally and internationally. The most reported causes of burnout syndrome were tension and lack of organization in teamwork, followed by lack of recognition from patients, supervisors, and colleagues, then demanding and challenging patients and family members. Overtime hours per month is independently associated with a higher risk of burnout, while non-Arab ethnicity is associated with a lower risk of burnout. Further in-depth studies are required to assess the underlying causes of burnout, and the risk in relation to social interactions, leadership involvement, and personal habits. The impact of burnout on medication errors should be evaluated as well.

## Supporting information

S1 Fig Flow chart of study participants. (DOCX)
