# Supplementary material for: Burnout among pharmacy professionals in Qatar: A cross-sectional study
Source: PLoS One. 2022 May 5;17(5):e0267438. doi: 10.1371/journal.pone.0267438 (PMC9071121; doi:10.1371/journal.pone.0267438)
Supplement: S1 Fig — (DOCX) [file pone.0267438.s001.docx]

**Fig: Flow chart of study participants**

MBI: Maslach Burnout Inventory
